# Abatement Cost of GHG Emissions for Wood-Based Electricity and Ethanol at Production and Consumption Levels

**DOI:** 10.1371/journal.pone.0100030

**Published:** 2014-06-17

**Authors:** Puneet Dwivedi, Madhu Khanna

**Affiliations:** 1 Warnell School of Forestry and Natural Resources, University of Georgia, Athens, Georgia, United States of America; 2 Energy Biosciences Institute, University of Illinois at Urbana-Champaign, Urbana, Illinois, United States of America; INSTITUTO MEXICANO DEL PETRÓLEO, Mexico

## Abstract

Woody feedstocks will play a critical role in meeting the demand for biomass-based energy products in the US. We developed an integrated model using comparable system boundaries and common set of assumptions to ascertain unit cost and greenhouse gas (GHG) intensity of electricity and ethanol derived from slash pine (*Pinus elliottii*) at the production and consumption levels by considering existing automobile technologies. We also calculated abatement cost of greenhouse gas (GHG) emissions with respect to comparable energy products derived from fossil fuels. The production cost of electricity derived using wood chips was at least cheaper by 1 ¢ MJ^−1^ over electricity derived from wood pellets. The production cost of ethanol without any income from cogenerated electricity was costlier by about 0.7 ¢ MJ^−1^ than ethanol with income from cogenerated electricity. The production cost of electricity derived from wood chips was cheaper by at least 0.7 ¢ MJ^−1^ than the energy equivalent cost of ethanol produced in presence of cogenerated electricity. The cost of using ethanol as a fuel in a flex-fuel vehicle was at least higher by 6 ¢ km^−1^ than a comparable electric vehicle. The GHG intensity of per km distance traveled in a flex-fuel vehicle was greater or lower than an electric vehicle running on electricity derived from wood chips depending on presence and absence of GHG credits related with co-generated electricity. A carbon tax of at least $7 Mg CO_2_e^−1^ and $30 Mg CO_2_e^−1^ is needed to promote wood-based electricity and ethanol production in the US, respectively. The range of abatement cost of GHG emissions is significantly dependent on the harvest age and selected baseline especially for electricity generation.

## Introduction

The electricity and transportation sectors of the US economy emitted 57% of total GHG emissions (6753 million Mg CO_2_e) in 2011 [1]. Therefore, policy makers have announced several incentives to promote electricity generation from various renewable sources including biomass to reduce GHG emissions from the electricity sector [2]. It is projected that these incentives will increase biomass-based electricity generation at the national level from 11.5 to 49.3 billion kWh between 2010 and 2035 [3]. There is an emphasis on reducing GHG emissions from the transportation sector as well. The Energy Independence and Security Act of 2007 has set a target of producing 60.5 billion liters of cellulosic biofuels by 2022 nationwide [4].

Biomass obtained from the nation’s forestlands would play a critical role in supplying required biomass for renewable electricity generation and production of cellulosic ethanol [5]. A few studies have analyzed economic and environmental potential of utilizing forest biomass for generating electricity [6]–[12] and producing ethanol [13]–[19]. Typically, these studies indicate that wood-based energy products could save significant amounts of GHG emissions (about 80% or more) but are costlier (at least 15% or more) than equivalent energy products derived from fossil fuels. These studies use different species, energy pathways, system boundaries, and modeling assumptions; therefore, it is practically very difficult to compare these studies with each other to get an insight about the cost-effectiveness of various woody feedstocks in reducing GHG emissions. No study has done a side-by-side comparison of the economic and environmental performance of wood-based electricity and ethanol at the production and consumption levels for existing automobile technologies using similar assumptions under realistic system boundaries. Comparable existing studies only focus on agriculture feedstocks and typically consider environmental [20]–[22] and economic performances [23], [24] of energy products disjointedly. A consideration of both economic and environmental performances of different bioenergy products in a single framework is critical to compare cost-effectiveness of various GHG mitigation options to minimize total cost related with the reduction of GHG emissions at the national and regional levels [25]. Additionally, these information will help in determining the minimum carbon tax that would be needed to promote production and consumption of wood-based energy products in the US. Furthermore, existing studies [6]–[19] measure economic and environmental performances of biomass-based energy products either at production or consumption levels but not at both levels simultaneously. This gives an incomplete picture as it is our assertion that the performance of biomass-based energy products could vary significantly at the selected level of analysis as fuel economy of automobiles operating on ethanol and electricity differ from each other [26].

We analyzed four energy pathways in this study. Focus of first two energy pathways was on electricity generation while the last two energy pathways focused on ethanol production. Under the first energy pathway, wood was converted to wood pellets and then manufactured wood pellets were burned at a nearby power plant to generate electricity. This pathway was based on the fact that the US has become a major exporter of wood pellets to power plants located in European countries [27]. We wanted to test the economic and environmental feasibility of utilizing manufactured wood pellets within the US only assuming that power plant owners in the country will follow a similar trend in the future as well. Under the second energy pathway, wood was chipped at the forest site and then wood chips were directly burned at a nearby power plant to generate electricity. For the third energy pathway, feedstock was chipped at the forest site and then sent to an ethanol mill for ethanol production [5]. The co-generated electricity was supplied to the grid for additional income and GHG credits. Under the fourth energy pathway, feedstock was chipped at the forest site and then sent to an ethanol mill for ethanol production [5]. However, co-generated electricity was not supplied to the grid and therefore, no additional income and GHG-credits were accrued.

For each energy pathway, we analyzed 186 scenarios (three feedstocks – logging residues only, pulpwood only, both logging residues and pulpwood; two forest management choices – intensive and non-intensive; 31 harvest ages – age 10 to age 40 in steps of 1 year). We selected pulpwood as a potential feedstock as evidence suggests that it is increasingly being used to manufacture wood pellets [27]. Under intensive forest management, herbicides were applied at the establishment year followed by fertilizers at plantation ages 2 and 12. No herbicides and fertilizers were applied under non-intensive forest management choice. Intensive forest management represents industrial plantations whereas non-intensive forest management represents plantation owned by non-industrial private forestland owners. The geographical focus of this study is US South as this region contributed about 62% of total roundwood removals in 2006 nationwide [28]. We selected slash pine as a representative species as this species is a popular commercial forest species of the region [28]. Additionally, pine plantations contribute maximum to the overall roundwood harvest in southern forestry landscape and therefore, focusing on a popular pine species will define the role of existing forest resources in the region in mitigating GHG emissions. This becomes even more important as the majority of existing studies focus on short rotation woody crops like willow [8], [11], [16], eucalyptus [18], and poplar [19].

## Methods

### Feedstock Availability

We used a growth and yield model of slash pine [29] to estimate availability of three timber products: sawtimber, chip-n-saw, and pulpwood under intensive and non-intensive forest management choices at different plantation years. The availability of logging residues at a plantation year was calculated as the difference between total biomass present in logs and total biomass present in merchantable portion of logs (sawtimber, chip-n-saw, and pulpwood) plus 20% of biomass present in sawtimber, chip-n-saw, and pulpwood at the same plantation year [30]. Additional 20% biomass was added as a proxy for biomass available in branches and tree tops [30].

### GHG Intensity of First Energy Pathway

We calculated total wood pellets produced (WP in Mg ha^−1^) at a harvest age using Equation (1).
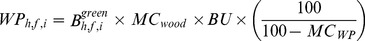
(1)where, B^green^ is the biomass available at a given harvest age (*h*), feedstock type (*f*), and forest management intensity (*i*); MC_wood_ is the moisture content of the green wood (50%); BU is the ratio of biomass used for wood pellet production (80%) [6]; and MC_WP_ is the moisture content of wood pellets (5%) [6]. We calculated total electricity generated (EC^WP^ in MJ ha^−1^) from wood pellets using Equation (2).
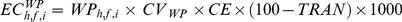
(2)where, CV_WP_ is the calorific value of wood pellets (18.5 MJ kg^−1^), CE is the conversion efficiency of a 100 MW power plant (31.70%) [31], and TRAN is the electricity transmission losses (7%) [32]. A 100 MW power plant is considered based on the fact that several large-scale facilities have recently been established in the US and Europe which will utilize wood pellets/wood chips to generate electricity [33], [34]. We calculated GHG intensity 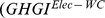
in g CO_2_e MJ^−1^) of generated electricity from wood pellets using Equation (3).
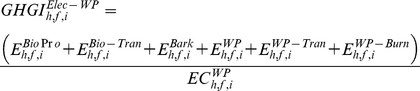
(3)where, E^Bi^°^Pr^° (Mg CO_2_e ha^−1^) represents GHG emissions related to wood production. The total GHG emission under intensive forest management was 4803 kg CO_2_e ha^−1^ when the harvest age was equal or greater than 12 years whereas it was 2431 kg CO_2_e ha^−1^ when the harvest age was 10 and 11 years [6]. For non-intensive forest management choice, total GHG emission was 2200 kg CO_2_e ha^−1^ for the selected range of harvest ages [6]. We updated the value of nitrous oxide emission based on the GREET model [35]. These GHG emissions were allocated to feedstocks based on the percentage of mass occupied by feedstocks out of total timber products available at a given harvest age. The parameter E^Bi^°^−Tran^ reflects GHG emissions related to transportation of biomass from a harvest site to a nearby wood pellet plant. It was a product of GHG emission factor (0.133 kg CO_2_e Mg^−1 ^km^−1^) [36], total green biomass transported, and average distance traveled (100 km one way). The parameters E^Bark^ reflects non-biogenic GHG emissions related with bark burning in a boiler (34.4 g CO_2_e kg^−1^ of burned material) [37]. Percentage of bark was 20% of incoming biomass [6]. The parameter E^WP^ reflects GHG emissions related with manufacturing of wood pellets (155.7 g CO_2_e kg^−1^) [6]. The parameter E^WP-Tran^ reflects GHG emissions related to transportation of wood pellets from wood pellet mill to a nearby power plant. It was a product of GHG emission factor (0.133 kg CO_2_e Mg^−1 ^km^−1^) [36], total wood pellets transported, and average distance traveled (50 km one way). We followed steps for estimating parameter E^Bark^ for quantifying non-biogenic GHG emissions related with the burning of wood pellets (E^WP-Burn^) at a power plant.

### GHG Intensity of Second Energy Pathway

We calculated total wood chips produced (WC in Mg ha^−1^) at a harvest age using Equation (4).
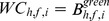
(4)


We calculated total electricity generated (EC^WC^ in MJ ha^−1^) from wood chips using Equation (5).
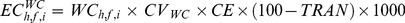
(5)where, CV_WC_ is the calorific value of wood chips (10 MJ kg^−1^). We calculated GHG intensity 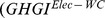 (in g CO_2_e MJ^−1^) of generated electricity from wood chips using Equation (6).
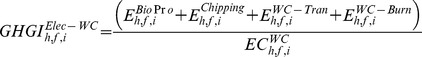
(6)where, the parameters E^chipping^ refers to GHG emissions related to chipping of feedstocks (E^Chipping^, 4 kg CO_2_e Mg^−1^) on the forest site [32] and E^WC–Burn^ reflects non-biogenic GHG emissions related with burning of wood chips in a boiler (34.4 g CO_2_e kg^−1^ of burned material) [37]. The parameter E^WC–Tran^ reflects GHG emissions related to transportation of wood chips from a harvest site to a nearby power plant. It was a product of GHG emission factor (0.133 kg CO_2_e Mg^−1 ^km^−1^) [36], total wood chips transported, and average distance traveled (100 km one way). The parameters E^WC–Burn^ reflects non-biogenic GHG emissions related with burning of wood chips in a boiler (34.4 g CO_2_e kg^−1^ of burned material) [37].

### GHG Intensity of Third Energy Pathway

Ethanol yield from a metric ton of bone dry feedstock was 329.6 l [38]. The conversion technology was assumed as dilute acid-pretreatment of feedstock followed by enzymatic hydrolysis [38]. The value of co-generated electricity at the time of ethanol production was 0.48 kWh l^−1^ of ethanol [38]. We multiplied total available biomass (B^green^) with the half of ethanol yield to estimate total ethanol availability (EE in l ha^−1^). We used Equation (7) to estimate the GHG intensity 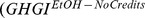 in g CO_2_e MJ^−1^) of ethanol.
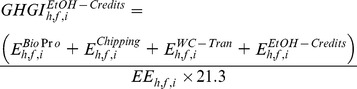
(7)where, E^EtOH–Credits^ refers to GHG emissions related to conversion of biomass into ethanol and transporting it to a nearby pump station (50 km one side). We obtained this value (–106.5 g CO_2_e l^−1^ or −5.0 g CO_2_e MJ^−1^ of ethanol produced) from the GREET model after updating default values of ethanol yield and co-generated electricity with values used in this study [35]. Calorific value of ethanol was 21.3 MJ l^−1^ [35].

### GHG Intensity of Fourth Energy Pathway

We used Equation (8) to estimate the GHG intensity 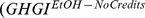 in g CO_2_e MJ^−1^) of ethanol.
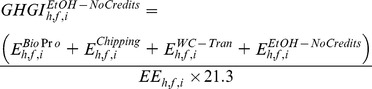
(8)where, E^EtOH–N^°^Credits^ refers to GHG emissions related to conversion of biomass into ethanol and transporting it to a nearby pump station (50 km one way). We obtained this value (191.7 g CO_2_e l^−1^ or 9.0 g CO_2_e MJ^−1^ of ethanol produced) from the GREET after updating default values of ethanol yield and co-generated electricity with values used in this study [35].

### Unit Cost Estimation

We calculated land expectation value (LEV in $ ha^−1^) at different harvest ages under intensive forest management using Equation (9). The LEV is defined as the net present value of bare forestland over infinite forest rotations [39]. We used parameters given in Table S1 in File S1 for calculating LEVs.
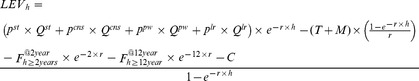
(9)where, p*^st^*, p*^cs^*, p*^pw^*, and p*^lr^* represent prices of sawtimber, chip-n-saw, pulpwood, and logging residues, respectively. Parameters Q*^st^*, Q*^cs^*, Q*^pw^*, and Q*^lr^* represent quantities of sawtimber, chip-n-saw, pulpwood, and logging residues available at a given harvest age, respectively. Parameters C, T, and M represent site preparation cost, annual taxes, and annual cost of plantation management, respectively. Parameter F represents cost of fertilizers applied at the 2^nd^ and 12^th^ year of plantation. Parameter *r* stands for the real discount rate (4%). We selected the highest LEV out of all LEVs and declared the corresponding harvest age as the optimal rotation age. Then, we subtracted LEVs for different harvest ages from the LEV at the optimal rotation age to determine the opportunity cost of changing harvest age. We made suitable changes in Equation (9) to ascertain LEVs at different harvest ages for non-intensive forest management. We have not considered the income obtained from logging residues while calculating LEVs for intensive and non-intensive forest management choices when they were not used as a feedstock. Similarly, we have not allocated any GHG emissions related to biomass production to logging residues when they were not used as a feedstock. Parameters reported in Table S2 in File S1 were used for ascertaining production cost of a MJ of generated electricity and produced ethanol.

### Abatement Cost

We used Equation (10) to estimate the abatement cost of a metric ton of GHG emission for both bioenergy products.
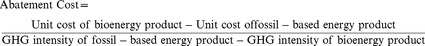
(10)


The units of numerator and denominator portions of the above equation were ¢ km^−1^ and g CO_2_e km^−1^, respectively. The fuel economies of an electric and flex-fuel vehicles were taken as 1.4 km MJ^−1^ and 0.35 km MJ^−1^, respectively [26]. The levelized unit production cost of electricity generated from coal and natural gas was taken as 2.78 ¢ MJ^−1^ and 1.87 ¢ MJ^−1^, respectively [40]. Levelized electricity generation costs for electricity derived from biomass, coal, and natural gas are based on new generation sources for 2018 expressed in 2011 dollars. The wholesale price of gasoline was taken as 2.56 ¢ MJ^−1^ [41]. The GHG intensity of electricity generated from coal and natural gas was taken as 343.1 g CO_2_e MJ^−1^ and 178.61 g CO_2_e MJ^−1^, respectively [20]. The GHG intensity of gasoline was taken as 94 g CO_2_e MJ^−1^ [35].

## Results

**Figure 1:**
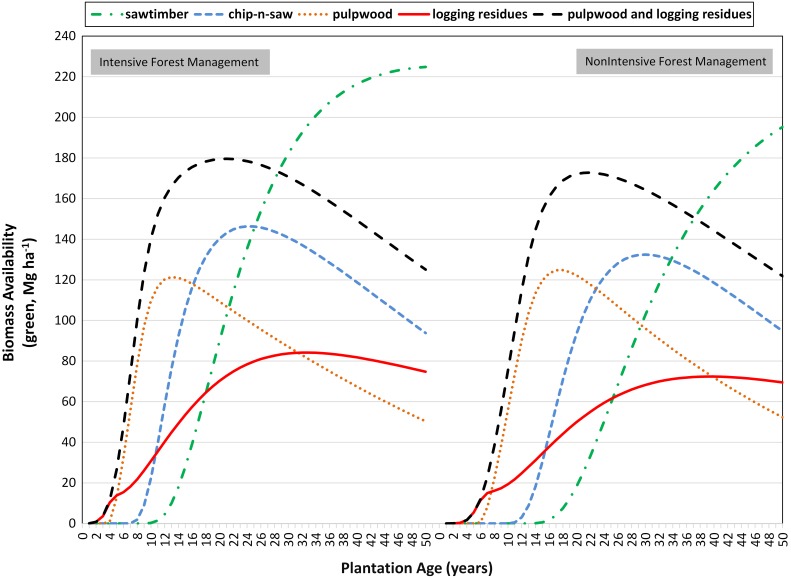
Availability of timber products at different plantation ages. Site index is 21.4 meters at 25^th^ year of plantation. Initial plantation density is 1236 seedlings ha^−1^.

The availability of large-diameter timber products (sawtimber and chip-n-saw) was smaller at initial harvest ages relative to small-diameter timber products (pulpwood and logging residues). However, availability of large-diameter timber products increased as trees gained girth and height with time (Figure 1). The availability of logging residues was maximum at harvest ages 33 (84.2 Mg ha^−1^) and 39 (72.4 Mg ha^−1^) years for intensive and non-intensive forest management, respectively. The availability of pulpwood was highest at harvest ages 13 (121.4 Mg ha^−1^) and 18 (124.8 Mg ha^−1^) years for intensive and non-intensive forest management, respectively. The combined availability of pulpwood and logging residues reached to a maximum value of 179.6 and 172.8 Mg ha^−1^ at plantation ages 21 and 22 years under intensive and non-intensive forest management scenarios, respectively. Total availability of logging residues was always higher under intensive than non-intensive forest management at all harvest ages. Same case was observed with the combined availability of logging residues and pulpwood. However, total availability of pulpwood was only higher under intensive than non-intensive forest management when harvest age was lower than 16 years.

**Figure 2:**
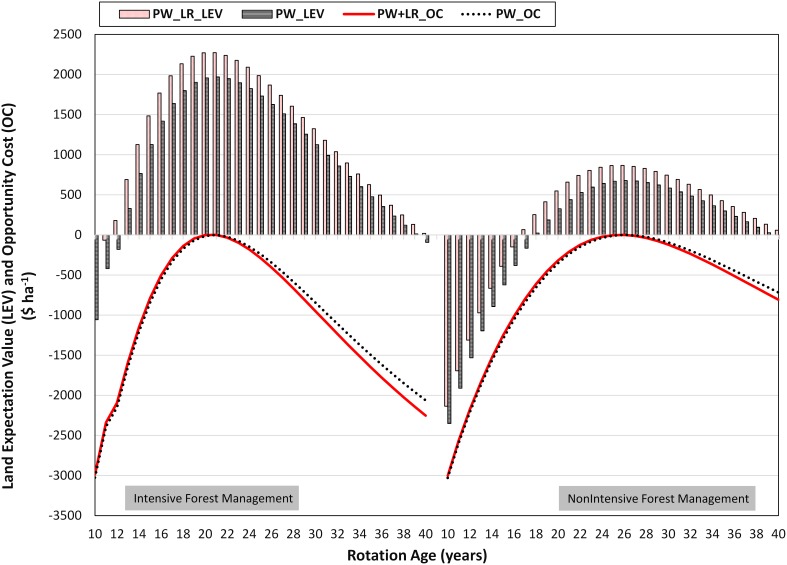
Distribution of land expectation values (LEVs) and opportunity costs (OCs). Opportunity cost is calculated by subtracting land expectation value at a given harvest age from the land expectation value at the optimal rotation age. The land expectation value is highest at the optimal rotation age.

**Figure 3:**
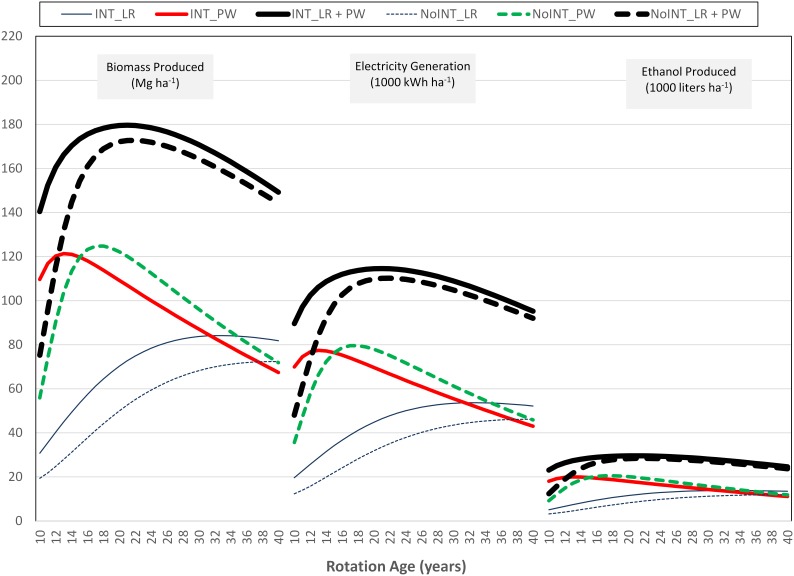
Availability of feedstocks, electricity generated, and ethanol produced. LR: logging residues; PW: pulpwood; INT: intensive forest management; NoINT: non-intensive forest management.

Under intensive and non-intensive forest management choices, LEVs were highest at 21^st^ and 26^th^ year of plantation, respectively (Figure 2). Thus, optimal rotation ages for intensive and non-intensive forest management choices were 21 and 26 years, respectively. Additional income from logging residues increased the LEV by 15 and 28 percentage points for intensive and non-intensive forest management choices at optimal rotation ages, respectively. As expected, opportunity cost increased with an increase or a decrease in the harvest age from the optimal rotation age. Quantities of total electricity generated and ethanol produced were proportional to the feedstock availability (Figure 3).

**Figure 4:**
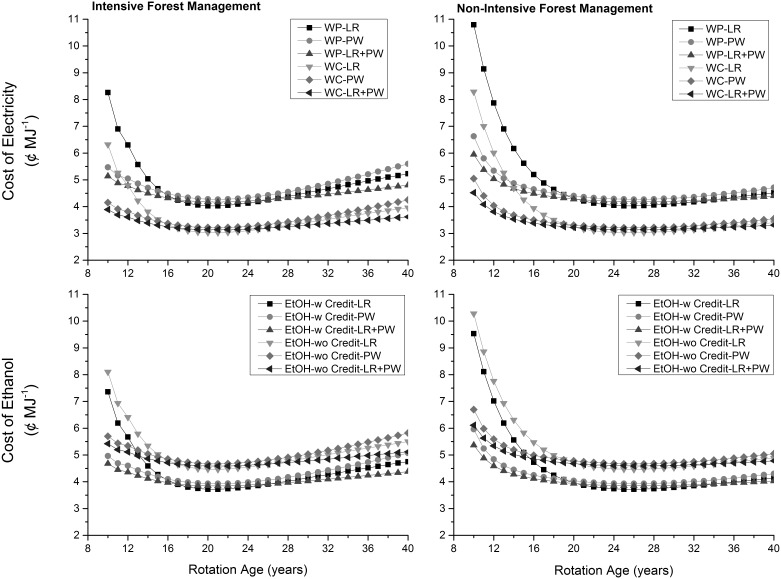
Cost of energy products at the production level. LR: logging residues; PW: pulpwood; WP: wood pellets; WC: wood chips; w: with income from cogenerated electricity; wo: without income from cogenerated electricity.

**Figure 5:**
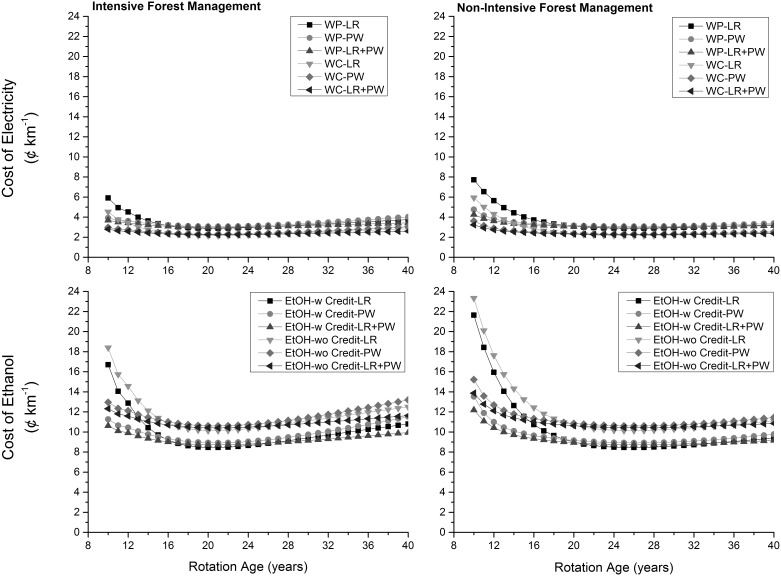
Cost of energy products at the consumption level. LR: logging residues; PW: pulpwood; WP: wood pellets; WC: wood chips; w: with income from cogenerated electricity; wo: without income from cogenerated electricity.

The cost of electricity generated from wood pellets was consistently higher (about 1.0 to 2.5 ¢ MJ^−1^) than the cost of electricity generated using wood chips across same feedstocks mostly due to higher production and transportation costs of wood pellets (Figure 4). The cost of ethanol produced without any income from co-generated electricity was higher by 0.7 ¢ MJ^−1^ than the cost of ethanol produced with income from co-generated electricity across same feedstocks. Across energy pathways, the cost of per MJ of energy obtained in the form of ethanol without any income from co-generated electricity was highest followed by electricity from wood pellets, ethanol with income from co-generated electricity, and electricity from wood chips. Unit production costs were comparable across feedstocks and choice of forest management especially after 12^th^ year of plantation. At the consumption level, the cost of a km traveled using electricity produced with wood pellets was higher than that of a km traveled with electricity generated from wood chips (0.7 to 1.8 ¢ km^−1^) across feedstocks (Figure 5). The cost of a km with ethanol produced in the presence of income from co-generated electricity was lower than the cost of a km with ethanol produced in the absence of income from co-generated electricity by 1.7 ¢ km^−1^. A comparison across energy pathways revealed that a km of travel was much cheaper for an electric vehicle than a flex-fuel vehicle ranging from 5.6 ¢ km^−1^ and 17.4 ¢ km^−1^ depending upon whether wood pellets or wood chips were used for electricity generation (Table 1). This was mostly due to high fuel economy of electric vehicles than flex fuel vehicles.

**Table 1:** Range of production costs and GHG intensities for selected energy pathways.

		Unit Cost												GHG Intensity											
		ProductionLevel (¢ MJ^−1^)						ConsumptionLevel (¢ km^−1^)						ProductionLevel (g CO_2_e MJ^−1^)						ConsumptionLevel (g CO_2_e km^−1^)					
		Intensive			Non-Intensive			Intensive			Non-Intensive			Intensive			Non-Intensive			Intensive			Non-Intensive		
		LR	PW	LR+PW	LR	PW	LR+PW	LR	PW	LR+PW	LR	PW	LR+PW	LR	PW	LR+PW	LR	PW	LR+PW	LR	PW	LR+PW	LR	PW	LR+PW
Electricity fromWood Pellets	Min	4.0	4.3	4.2	4.0	4.3	4.2	2.9	3.1	3.0	2.9	3.1	3.0	49.0	49.0	47.1	47.1	47.1	20.8	35.1	35.7	35.1	33.7	34.0	33.7
	Max	8.3	5.6	5.1	10.8	6.6	5.9	5.9	4.0	3.7	7.7	4.7	4.3	54.2	56.2	54.2	57.6	62.0	57.6	38.8	40.3	38.8	41.2	44.4	41.2
Electricity fromWood Chips	Min	3.0	3.2	3.1	3.0	3.2	3.1	2.2	2.3	2.2	2.2	2.3	2.2	20.8	21.5	20.8	19.3	19.6	19.3	14.9	15.4	14.9	13.8	14.0	13.8
	Max	6.3	4.2	3.9	8.3	5.0	4.5	4.5	3.0	2.8	5.9	3.6	3.2	24.8	26.4	24.8	27.4	30.9	27.4	17.8	18.9	17.8	19.6	22.1	19.6
Ethanol withco-generatedelectricity	Min	3.7	3.9	3.8	3.7	3.9	3.9	8.4	8.9	8.7	8.4	8.9	8.7	3.5	4.3	3.5	1.8	2.2	1.8	8.0	9.7	8.0	4.2	5.1	4.2
	Max	7.4	5.1	4.7	9.5	6.0	5.4	16.7	11.5	10.6	21.6	13.5	12.2	7.9	9.7	7.9	10.9	14.6	10.9	18.0	22.1	18.0	24.6	33.2	24.6
Ethanol withoutco-generated electricity	Min	4.5	4.7	4.6	4.5	4.7	4.6	10.1	10.6	10.4	10.1	10.6	10.4	21.9	22.7	21.9	20.2	20.6	20.2	49.7	51.4	49.7	45.9	46.8	45.9
	Max	8.1	5.8	5.4	10.3	6.7	6.1	18.4	13.2	12.3	23.3	15.2	13.9	26.3	28.1	26.3	29.2	33.0	29.2	59.8	63.8	59.8	66.4	75.0	66.4

LR: logging residues; PW: pulpwood; Intensive: intensive forest management; Non-intensive: non-intensive forest management.

**Figure 6:**
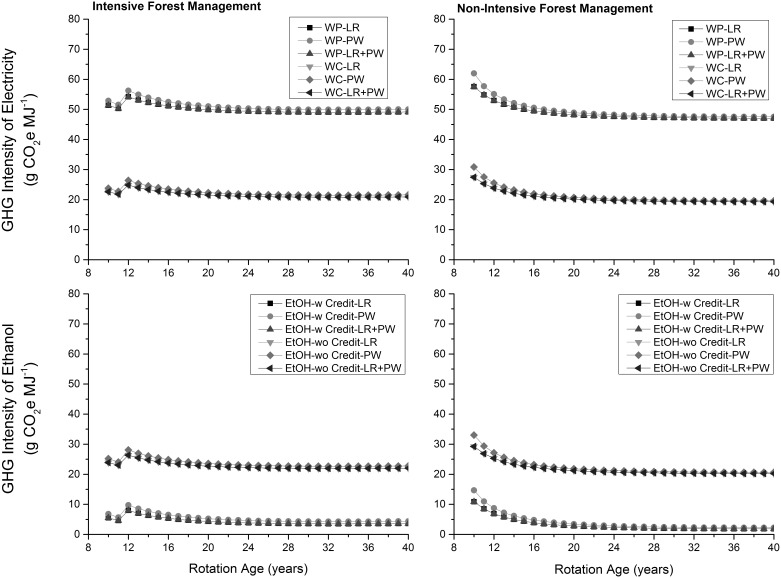
GHG intensity of energy products at the production level. LR: logging residues; PW: pulpwood; WP: wood pellets; WC: wood chips; w: with income from cogenerated electricity; wo: without income from cogenerated electricity.

**Figure 7:**
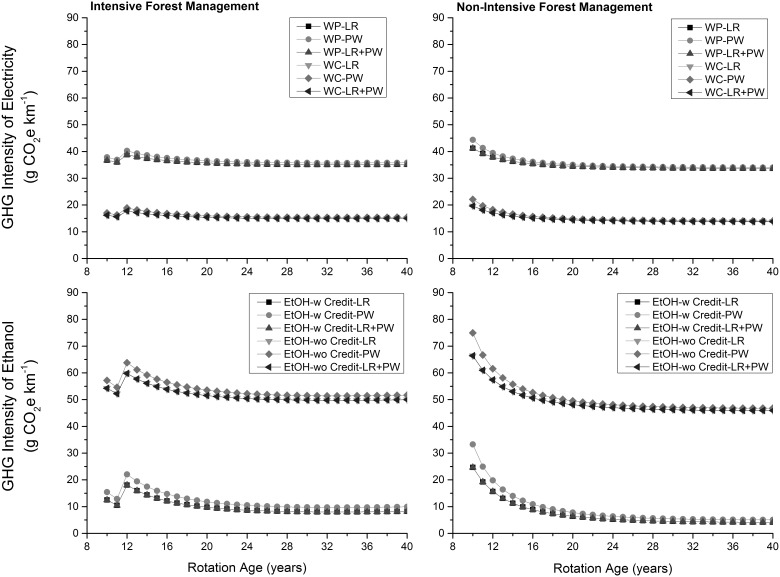
GHG intensity of energy products at the consumption level. LR: logging residues; PW: pulpwood; WP: wood pellets; WC: wood chips; w: with income from cogenerated electricity; wo: without income from cogenerated electricity.

**Figure 8:**
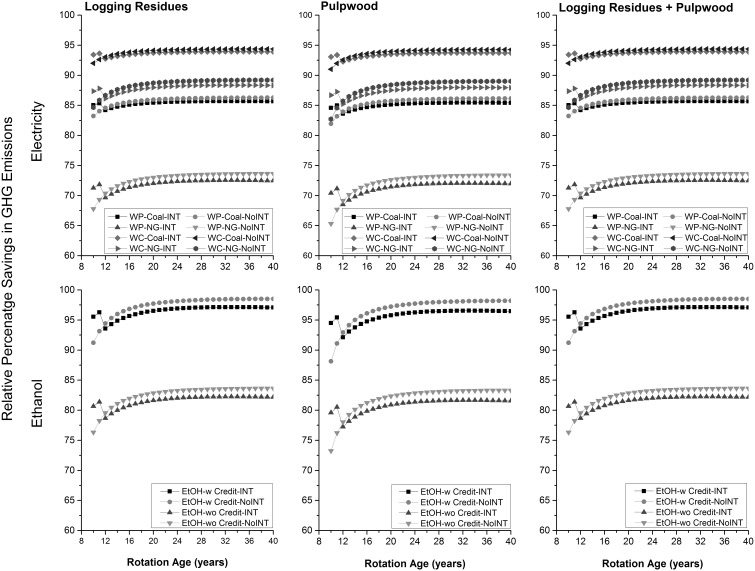
Relative percentage savings in GHG emissions. LR: logging residues; PW: pulpwood; WP: wood pellets; WC: wood chips; w: with income from cogenerated electricity; wo: without income from cogenerated electricity.

The GHG intensity of electricity generated from wood pellets was highest whereas the GHG intensity of ethanol produced in presence of GHG credits due to supply of co-generated electricity to the grid was lowest at the production level (Figure 6). The GHG intensities of electricity generated from wood chips and ethanol produced in absence of any GHG credits were comparable at the production level (Table 1). At the consumption level, the GHG intensity of ethanol produced in the absence of any GHG credits was highest followed by electricity generated using wood pellets, electricity generated from wood chips, and ethanol produced in the presence of GHG credits (Figure 7). Percentage savings in GHG emissions relative to the electricity generated from coal and natural gas on per km traveled across feedstocks remained almost same (Figure 8). This was also the case for the produced ethanol. For generated electricity, relative percentage savings were higher (about 8% and 15% relative to coal and natural gas, respectively) when wood chips were used as a feedstock than wood pellets. Similarly, relative percentage savings were higher (about 15%) when GHG credits from co-generated electricity were considered. Across forest management choices, percentage savings in GHG emissions for non-intensive than intensive forest management were higher by about 2% only.

**Figure 9:**
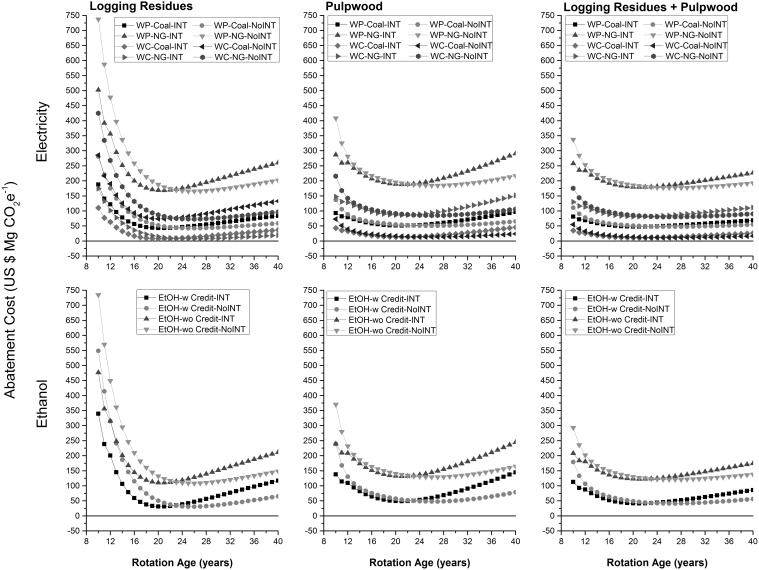
Abatement cost of GHG emissions with respect to corresponding fossil fuel-based energy products. LR: logging residues; PW: pulpwood; WP: wood pellets; WC: wood chips; w: with income from cogenerated electricity; wo: without income from cogenerated electricity.

For generated electricity and produced ethanol, the abatement cost of GHG emissions did not vary much across feedstocks (Figure 9). Based on lowest abatement cost, a minimum carbon tax of $ 7.7 Mg CO_2_e^−1^ or $ 73 Mg CO_2_e^−1^ would be required to promote production of electricity from wood chips with respect to electricity generated using coal and natural gas, respectively (Table 2). A minimum carbon tax of $ 42.5 Mg CO_2_e^−1^ or $ 165 Mg CO_2_e^−1^ would be required to promote production of electricity from wood pellets with respect to electricity generated using coal and natural gas, respectively. Similarly, a minimum carbon tax of $ 31 Mg CO_2_e^−1^ or $ 108 Mg CO_2_e^−1^ would be required to promote wood-based ethanol depending upon whether or not income and GHG credits from co-generated electricity at the time of ethanol production were considered. The abatement cost was higher under non-intensive than intensive forest management before harvest age of 24 years but for harvest ages 24 years and greater, the abatement cost was higher under intensive than non-intensive forest management. For generated electricity, the abatement cost was at least $ 34.8 Mg CO_2_e^−1^ and $ 92.3 Mg CO_2_e^−1^ less when wood chips were used as a fuel than wood pellets with respect to electricity generated using coal and natural gas, respectively. Relative abatement cost was at least $ 70 Mg CO_2_e^−1^ less for ethanol produced in presence of income and GHG credits due to co-generated electricity than in absence of them.

**Table 2:** Range of abating GHG emissions for selected energy pathways.

		Abatement Cost($ Mg^−1^ CO2e)(electricity relative to coal)						Abatement Cost($ Mg^−1^ CO2e)(electricity relative to natural gas)						Abatement Cost($ Mg^−1^ CO2e)(ethanol relative to gasoline)					
		Intensive			Non-Intensive			Intensive			Non-Intensive			Intensive			Non-Intensive		
		LR	PW	LR+PW	LR	PW	LR+PW	LR	PW	LR+PW	LR	PW	LR+PW	LR	PW	LR+PW	LR	PW	LR+PW
Electricity fromWood Pellets	Min	42.8	51.1	47.6	42.5	50.6	47.5	168.3	188.3	179.3	165.4	184.3	176.7	-	-	-	-	-	-
	Max	187.8	96.4	81.1	280.5	137.0	111.0	339.5	144.6	112.6	549.0	239.4	179.2	-	-	-	-	-	-
Electricity fromWood Chips	Min	7.7	13.5	11.1	7.7	13.4	11.3	73.9	86.1	80.9	73.0	85.0	80.4	-	-	-	-	-	-
	Max	110.4	45.8	34.8	174.5	72.7	55.1	285.3	151.9	130.0	424.9	215.6	175.5	-	-	-	-	-	-
Ethanol withco-generatedelectricity	Min	-	-	-	-	-	-	-	-	-	-	-	-	31.1	48.7	41.3	30.6	47.7	41.2
	Max	-	-	-	-	-	-	-	-	-	-	-	-	339.5	144.6	112.6	549.0	239.4	179.2
Ethanol withoutco-generatedelectricity	Min	-	-	-	-	-	-	-	-	-	-	-	-	110.3	131.8	122.5	108.2	128.8	120.7
	Max	-	-	-	-	-	-	-	-	-	-	-	-	476.8	244.8	207.9	735.0	370.3	293.0

LR: logging residues; PW: pulpwood; Intensive: intensive forest management; Non-intensive: non-intensive forest management.

## Discussion and Conclusions

The use of wood chips instead of wood pellets for electricity generation was a better option both in terms of unit cost and environmental performance in the US. This was mostly due to additional costs and GHG emissions related with the production and transportation of wood pellets. An abatement cost of electricity generated using woody feedstocks varied decisively depending upon the selected baseline of electricity generated from fossil fuels. Cost of abating GHG emissions by electricity produced from either wood pellets or wood chips was much lower when it replaces coal-based electricity than natural gas-based electricity. Income and GHG credits accrued due to the supply of co-generated electricity at the time of ethanol production played a critical role in determining unit cost and GHG intensity of produced ethanol. This implies that industrial operations at an ethanol mill should be optimized so that a certain portion of co-generated electricity is supplied to the grid to earn extra income and GHG credits.

Cost of driving a km of an electric vehicle using electricity generated from wood chips was cheaper than a comparative flex-fuel vehicle utilizing ethanol derived from same woody feedstocks. Similarly, the GHG intensity of covering a km of distance by an electric vehicle was less than a comparative flex-fuel vehicle running on ethanol derived in absence of any co-generated electricity. The GHG intensity was higher for a km of distance covered by electric vehicle utilizing electricity generated using wood chips or wood pellets than a km of distance covered by flex-fuel vehicle using ethanol produced in presence of GHG credits related to co-generated electricity. Overall this implies that use of an electric vehicle running on electricity derived from wood chips should be preferred for simultaneously maximizing environmental and economic efficiencies. However, the abatement cost of doing so could range from $7 to $425 Mg CO_2_e^−1^ depending upon the selected baseline of electricity generated from fossil fuels and the harvest age. We also found that the minimum abatement cost of GHG emissions for electricity derived from wood pellets (with respect to coal-based electricity) and ethanol derived in presence of co-generated electricity were close to each other especially for rotation ages which were near to optimal rotation ages.

The opportunity cost related with a change in rotation age from the optimal rotation age was a significant determinant of unit production cost of wood-based energy products. A departure from the optimal rotation age increased the unit production cost of wood-based energy products implying that a significant change in rotation age from current rotation ages would increase the prices of wood-based energy products. The unit production cost and environmental performance of wood-based energy products did not vary across feedstocks. Therefore, logging residues and pulpwood can be used as individual feedstocks on their own for manufacturing of wood-based energy products. However, it is preferable to use both pulpwood and logging residues as a single feedstock from the perspective of land-use efficiency [21]. Relative savings in GHG emissions were about 2% higher under non-intensive than intensive forest management starting from 12^th^ year of plantation age implying that feedstocks derived from both intensive and non-intensive forest management could be used for wood-based bioenergy development without any significant drop in relative savings of GHG emissions.

This study suggests that the GHG intensity of wood-based energy products is less than the GHG intensity of corresponding fossil-fuel energy products. However, the unit production cost of wood-based energy products is higher than the corresponding fossil-fuel energy products depending upon the harvest age. This implies that financial support is required to promote production of wood-based energy products. This financial support could be in the form carbon tax on corresponding fossil fuel-based energy products. Other mechanism like subsidies/carbon markets should also be explored.

We have not considered carbon sequestered in soils in this study as carbon sequestered on reforested lands remain very stable with respect to time [42]. We have not considered carbon sequestered in other pools (live trees, dead trees, debris, and coarse roots) as well. We acknowledge this as a limitation of the existing study as a change in the rotation age will affect both these carbon pools with respect to time. A need exists to integrate the model developed in this study with national or regional economic-wide equilibrium models [43], [44] to assess the price dynamics of energy products derived from woody feedstocks with respect to the corresponding fossil-fuel energy products. This will also give an estimate of an opportunity cost related to diversion of pulpwood for bioenergy development than paper-based products. Moreover, we have primarily focused on variability in availability of feedstocks in this study. A need exists to capture variability on production technologies as well including other energy products like biodiesel and vehicle types. Finally, we have not considered biogenic emissions due to consumption of wood-based energy products as quantities of carbon at the landscape level under continuous forestry assumption does not change over time. We hope that this study will significantly benefit future research exploring carbon benefits of bioenergy development in the US and beyond. We are also hopeful that this study will provide policy makers an understanding about possible pathways and potential incentives needed to promote bioenergy development in the US.

## Supporting Information

File S1 Supporting tables.
(DOCX)
