# Supplementary material for: Abatement Cost of GHG Emissions for Wood-Based Electricity and Ethanol at Production and Consumption Levels
Source: PLoS One. 2014 Jun 17;9(6):e100030. doi: 10.1371/journal.pone.0100030 (PMC4061057; doi:10.1371/journal.pone.0100030)
Supplement: File S1 — Supporting tables. (DOCX) [file pone.0100030.s001.docx]

**Table S1:** Income and cost parameters used for estimating land expectation values. Site preparation includes shearing, raking, piling, burning, and bedding. Prices of logging residues are half of pulpwood prices. Initial plantation density is 1235 seedlings ha^-1^ and site index is 21.4 m at 25^th^ year of plantation.

| Income | | | |
| --- | --- | --- | --- |
| sawtimber | 29.7 [4] | $ Mg^-1^ | Intensive/non-intensive forest management choices. |
| chip-n-saw | 16.7 [4] | $ Mg^-1^ | Intensive/non-intensive forest management choices. |
| pulpwood | 10.96 [4] | $ Mg^-1^ | Intensive/non-intensive forest management choices. |
| logging residues | 5.4 [4] | $ Mg^-1^ | Intensive/non-intensive forest management choices. |
| Cost | | | |
| Tax | 7.4 [4] | $ ha^-1^ yr^-1^ | Intensive/non-intensive forest management choices. |
| Management | 25.9 [4] | $ ha^-1^ yr^-1^ | Intensive/non-intensive forest management choices. |
| Site preparation | 997.8 [4] | $ ha^-1^ | For non-intensive forest management: $911.3/ha. |
| Planting | 172.9 [4] | $ ha^-1^ | Intensive/non-intensive forest management choices. |
| Herbicide treatment | 86.5 [4] | $ ha^-1^ | Only applicable for intensive forest management. |
| Fertilization @ year 2 | 148.3 [4] | $ ha^-1^ | Only applicable for intensive forest management. |
| Fertilization @ year 12 | 197.6 [4] | $ ha^-1^ | Only applicable for intensive forest management. |

**Table S2:** Cost parameters used for estimating production cost of generated electricity and produced ethanol. The annual capacity of an ethanol mill is 291 million liters of ethanol [38]. Unit production cost of ethanol produced in this study is based on this capacity only.

| Cost Points | Values | Units |
| --- | --- | --- |
| Stumpage price of pulpwood | 10.96 [4] | $ Mg^-1^ |
| Stumpage price of logging residues | 5.48 [4] | $ Mg^-1^ |
| Chipping cost | 7.16 [1] | $ Mg^-1^ |
| Logging cost of feedstock | 10.47 [4] | $ Mg^-1^ |
| Transportation of feedstock | 0.096 [4] | $ Mg^-1^ km^-1^ |
| Conversion of feedstock to wood pellets | 50 [5] | $ Mg^-1^ of wood pellets |
| Transportation of wood pellets | 0.096 [4] | $ Mg^-1^ km^-1^ |
| Electricity production at the power plant from wood pellets | 6.9 [3] | ¢ kWh^-1^ |
| Electricity production at the power plant from wood chips | 6.9 [3] | ¢ kWh^-1^ |
| Conversion of feedstock to ethanol with co-generated electricity | 0.401 [2] | $ l^-1^ |
| Conversion of feedstock to ethanol without co-generated electricity | 0.52 [2] | $ l^-1^ |
| Transportation of ethanol | 0.096 [4] | $ Mg^-1^km^-1^ |

**References**

1. ORNL (2011) U.S. Billion-Ton Update: Biomass Supply for a Bioenergy and Bioproducts Industry. Oak Ridge National Laboratory. Oak Ridge, TN.
2. Humbird D, Davis R, Tao L, Kinchin C, Hsu D, et al. (2011) Process Design and Economics for Biochemical Conversion of Lignocellulosic Biomass to Ethanol: Dilute-Acid Pretreatment and Enzymatic Hydrolysis of Corn Stover. National Renewable Energy Laboratory. Golden, CO.
3. USEIA (2012) Levelized Cost of New Generation Resoruces. United States Energy Information Adminstration. Washington, DC. Available: <http://www.eia.gov/forecasts/aeo/er/electricity_generation.cfm>.
4. TMS (2013) Southeastern Average Stumpage Prices. Timber Mart South, Warnell School of Forestry and Natural Resoruces, University of Georgia.
5. Qian, Y., McDow, W. (2013) The Wood Pellet Value Chain: An Economic Analysis of the Wood Pellet Supply Chain from the Southeast United States to European Consumers. U.S. Endowment for Forestry & Communities, Inc. Greenville, SC.
